# Effect of Gabapentin on Morphine Consumption and Pain after Surgical Debridement of Burn Wounds: A Double-Blind Randomized Clinical Trial Study

**DOI:** 10.5812/atr.5304

**Published:** 2012-06-01

**Authors:** Siamak Rimaz, Cyrus Emir Alavi, Abbas Sedighinejad, Mohammad Tolouie, Sharareh Kavoosi, Leila Koochakinejad

**Affiliations:** 1 Anesthesiology Research Center, Velayat University Hospital, Guilan University of Medical Sciences, Rasht, IR Iran; 2 Department of Burn Surgery, Velayat University Hospital, Guilan University of Medical Sciences, Rasht, IR Iran; 3 Department of Guilan Country Planning, Rasht, IR Iran; 4 Department of Nursing, Velayat University Hospital, Rasht, IR Iran

**Keywords:** Surgery, Postoperative Pain, Gabapentin, Morphine

## Abstract

**Background:**

Burn pain is recognized as being maximal during therapeutic procedures, and wound debridement can be more painful than the burn injury itself. Uncontrolled acute burn pain increases the stress response and the incidence of chronic pain and associated depression. Although opiates are excellent analgesics, they do not effectively prevent central sensitization to pain. The anticonvulsant gabapentin has been proven effective for treating neuropathic pain in large placebo-controlled clinical trials. Experimental and clinical studies have demonstrated antihyperalgesic effects in models with central neuronal sensitization. It has been suggested that central neuronal sensitization may play an important role in postoperative pain.

**Objectives:**

The aim of this study was to investigate the effect of gabapentin on morphine consumption and postoperative pain in burn patients undergoing resection of burn wounds.

**Patients and Methods:**

In a randomized, double-blind, placebo-controlled study, 50 burn patients received a single oral dose of gabapentin (1200mg) or placebo 2h before surgery. Anesthesia was induced with propofol and fentanyl and maintained by infusingpropofol, remifentanil, and 50% N2O in O2. All patients received patient-controlled analgesia with morphine at doses of 2.5 mg bolus and a lock-out time of 10 min for 24h before the operation. Pain was assessed on a visual analog scale (VAS) at rest and during movement at 1,4,8,12,16,20, and 24 h before the operation. Heart rate, oxygen saturation, mean arterial blood pressure, respiratory rate, sedation score, and morphine consumption were studied.

**Results:**

All the enrolled patients were able to complete the study; therefore, data from 50 patients wereanalyzed. The VAS scores at rest andduring movement at 1,4,8,12,16,20, and 24 h after the operation were significantly lower in the gabapentin group than in the placebo group (P < 0.05). Morphine consumption was significantly lessr in the gabapentin group than in the placebo group (P < 0.05). Sedation scores were similar in the 2 groups at all measured times. There were no differences in adverse effects between the groups.

**Conclusions:**

A single oral dose of 1200mg gabapentin resulted in a substantial reduction in postoperative morphine consumption and pain scores after surgical debridement in burn patients.

## 1. Background

All burn injuries are painful, and burn patients commonly experience high levels of acute and excruciating pain during hospitalization. Pain is perceived at the time and site of the burn because of the stimulation of local nociceptors and transmission of nerve impulse in the Aδ and C fibers. These impulses relay the pain message to the dorsal horn of the spinal cord. Conscious perception of pain occurs as the impulse is transmitted onwards to the brain and into areas collectively known as the pain matrix (1). Compbell *et al. *(1984), indicated that the burn injury resulted inincreased sensitivity of Afibers, decreased sensitivity of C fibers, increased pain sensitivity (hyperalgesia), and difficult-to-treat excessive pain (2). Some studies have suggested that burn patients have increased levels of anxiety, especially relating to treatment and outcome, and that these levels may increase overtime (3, 4). Burn pain is recognized as being maximal during therapeutic procedures (5), and wound debridement can be more painful than the burn injury itself (6).Uncontrolled acute burn pain increases the incidence of chronic pain and associated depression (7), and correlates with suicidal ideation at the time of discharge from hospital (8). Post-traumatic stress disorder is a notable sequelae of major burns and is linked, both as a cause and as an effect, with poorly controlled burn pain (9, 10). Avariety of pain measurement techniques have been used with adult burn patients. One of the most common measures is the numeric scale (i.e., rating pain on a scale of 0–100). This scale measures the sensory component of a patient’s pain. The numeric scale is quick and easy to use because patients do not require a visual representation of the scale. To control background and procedural pain during the acute phase of burn treatment (72 h to 3 or 5 weeks, until the wounds are closed), the use of patient-controlled analgesia (PCA) is far superior to the PRN (from the Latin “Pro re nata” meaning“as needed”) method (11). Furthermore, PCA has been described as safe and effective in both adults and children (5, 12, 13). Of the agents recommended for the relief of acute pain, morphine has continued to be the mainstay for pain management. However, morphine binds to more than 1 receptor class, and morphine consumption is associated with side effects, such as respiratory depression, itching, nausea, vomiting, and constipation, that limit its use.An additional problem is the rapid development of tolerance to morphine and poor pain control (11). Tolerance is defined as a requirement of increasing doses of a particular opioid to achieve the same analgesic effect and applies to side effects too (14). Tolerance to morphine is associated with the activation of the central glutamate pathways and hyperalgesia. Using other compounds such as gabapentin and ketamine, which block* N*-methyl-D-aspartate (NMDA) receptor pathways, improves the management of pain that can no longer be controlled with morphine (15). Because of the multiplicity of mechanisms involved in postoperative pain, a multimodal analgesia regimen, with a combination of opioid and non-opioid analgesic drugs, is often used to enhance analgesic efficacy and reduce opioid requirements and side effects (16). Gabapentin, a structural analog of gamma-aminobutyricacid, has antinociceptive and antihyperalgesic properties. It is known to bind to presynaptic calcium channels involved in pain hypersensitivity andto indirectly inhibit NMDA receptor overactivation (17). This explains the drug’s ability to limit long-term pain sensitization (18). Gabapentin and morphine have synergistic analgesic effects in animals and humans (19-21). In a recent study, a single dose of oral gabapentin reduced postoperative morphine consumption and pain after radical mastectomy (22). A small, retrospectivelymatched, case-controlled study showed a reduction in morphine requirement in acute burn patients following the administration of gabapentin for a 3-week period, commencing on day 3 after the burn (23).

## 2. Objectives

The aim of the present study was to determine the effect of a single preoperative oral dose of gabapentin on postprocedural and background pain and on PCA morphine consumption in burn patients after surgical resection of burn wounds. 

## 3. Patients and Methods

After obtaining approval from the hospital ethical committee and obtaining written informed consent from all participants, 50 patients undergoing surgical debridement of burn wounds were enrolled in the study. Inclusion criteria were as follows: patients had to have an American Society of Anesthesiologists (ASA) physical status classification of I or II (I = a normal healthy patient and II= a patient with mild systemic disease), had to be of age between 20 and 60 years, and had to have 10–35% total body surface area (TBSA) burns in the lower limbs. Patients were not included if they could not operate a PCA device, were breast feeding, had an allergy to gabapentin or morphine, or had a history of alcohol or drug abuse, chronic pain, daily intake of analgesics or anticonvulsants, antidepressant use, diabetes, or impaired kidney function. The study was conducted using a double-blind randomized controlled design, and all the operations were performed by the same surgeon. All patients were informed, during the pre-anesthetic examination, aboutthe use of the PCA device and the visual analog scale (VAS), in which 0 (zero) mm represented no pain and 100mm represented the worst pain imaginable. Patients were randomized, using a closed-envelope system, to receive either 1200 mg oral gabapentin (n=25) or placebo (n=25) 2 h before surgery. In the placebo group, patients were given placebo pills that were identical in appearance to gabapentin. Although the study drug (s) was given by a nurse, the investigators remained blinded to the group assignment. Upon arrival to the operating room, all patients were premedicated with 0.04 mg/kg of intravenous (IV) midazolam. Electrocardiographic electrodes and a non-invasive blood pressure monitor were applied and oxygen saturation (SpO_2_) was monitored by pulse oximetry. Anesthesia was induced with 2mg/kg propofol, 3µg/kg fentanyl, and 0.5 mg/kg atracurium. After endotracheal intubation, anesthesia was maintained with propofol infusion at a rate of 50 to 150 µg/kg/min with a fixed infusion of 0.4 µg/kg/min remifentanil, and 50% N_2_O in O_2_. The infusion of propofol and remifentanil was continued until the debridement of the burn wound was completed. Neuromuscular blockade was then reversed with IVadministration of 0.04 mg/kg neostigmine and 0.02 mg/kg atropine. After tracheal extubation, patients were transferred to the postanesthesia care unit (PACU). Postoperative analgesia was provided with IV-PCA morphine (2.5 mg bolus and 10 min lock-out time)upon arrival in the PACU. An additional 2.5 mg of morphine was administered intravenously by a nurse observer, if requested by the patient during the lock-out period. During the first 1h in the PACU, and then at 4, 8, 12, 16, 20, and 24 hours after extubation, patients were evaluated for pain scores (VAS) at rest and during mobilization from the supine to the sitting position. total dose of morphine consumption, heart rate (HR), SpO_2_,mean arterial pressure (MAP), respiratory rate (RR), and sedation score were also evaluated by an anesthesiology resident not otherwise involved in the study. The occurrence of any side effects, such as nausea, vomiting, constipation, respiratory depression, dizziness, somnolence, diarrhea, headache, and pruritus, was recorded. Morphine was stopped if the patient had a RR < 10 breaths/min, SpO_2_< 94% by pulse oximetry, or a serious adverse event related to opioid administration. Ondansetron (4mg) was intravenously administered for nausea and vomiting upon patient request. No other medications were administered during the 24-h observation period. A sample size of 25 patients per group was calculated to detect a significant difference of 15% or more in morphine consumption with a power of 85% and a significance level of 5%. Descriptive statistics are expressed as mean ± standard deviation (SD) unless otherwise stated. Normality of distribution was tested by the Kolmogorov–Smirnovtest. Student’s* t*-test was used for comparing the means of continuous variables and normally distributed data. The Mann–Whitney *U*-test was used in other cases. Two-way analysis of variance was used to compare groups, and Bonferroni correction and Tukey’s honestly significant difference (HSD) test were used for multiple comparisons. Categorical data were analyzed using eitherthe χ² testor Fisher’s exact test, as appropriate. Significance was determined at *P* < 0.05.

## 4. Results

From June1, 2009 to June 1, 2010, 50 consecutive patients who fulfilled the inclusion criteria were included in the study. All enrolled patients were able to complete the study; therefore, data from 50 patients were analyzed. The groups were comparable with respect to age, body weight, height, ASA physical status, duration of surgery, and burn size (*Table 1*). MAP, HR, SpO_2_, and respiratory rate were not different between the groups at any of the measured times (*Table 2*). Patients in the placebo group experienced more pain than the patients in the gabapentin group in both the lying and moving positions (*P *< 0.05) during the 24-h period after the debridement procedure (*Table 3*). Sedation scores were similar at all measured times in the gabapentin and placebo groups (*P *> 0.05). Postoperative morphine consumption at 8, 12, 16, 20, and 24h after surgerywas significantly lower in the gabapentin group than in the placebo group (*P *< 0.05) (*Table 4*). Total morphine consumption was reduced from 52.45 ± 10.4 mg in the placebo group to 33.8 ± 18mg in the gabapentin group (*P *< 0.05). The incidence of side effects is shown in *Table 5*. The most common side effects during the study were nausea and vomiting, but there was no difference in the incidence of side effects between the groups. Two patients in each group vomited, and 8 patients in the gabapentin group and 9 patients in the placebo group received ondansetron (*P *> 0.05). 

**Table 1.** Demographic and Clinical Data of the Patients

	Placebo (n=25) ^b^	Gabapentin (n=25) ^b^
Age, y, Mean ± SD ^a^	48.4 ± 10.2	50.5 ± 11.2
Weight, kg, Mean ± SD ^a^	70.5 ± 12	71.1 ± 14.4
Gender		
Male	8	7
Female	17	18
ASA Physical status ^a^		
I	20	21
II	5	4
Duration of anesthesia, min, Mean ± SD ^a^	23 ± 67	21 ± 59
Burn size, %, Mean ± SD ^a^	13 ± 25	19 ± 29

^a^Abbreviations: ASA, American Society of Anesthesiologists; SD, Standard Deviation

^b^No significant differences were found between the groups

**Table 2.** Differences in Postoperative Heart Rate, Mean Arterial Blood Pressure, and Respiratory Rate Between the Groups

	Placebo (n=25) ^b^	Gabapentin (n=25) ^b^
0, h		
HR ^a^, beats/min, Mean ± SD ^a^	99.2 ± 18.3	99.9 ± 17.8
MAP ^a^, mm Hg, Mean ± SD	113 ± 22.1	109.1 ± 21.1
RR ^a^, breaths/min, Mean ± SD	18.3 ± 2	17.4 ± 1.9
1, h		
HR , beats/min, Mean ± SD	97.6 ± 17.9	95.1 ± 16.8
MAP, mm Hg, Mean ± SD	106 ± 21	105 ± 19.3
RR, breaths/min, Mean ± SD	18.2 ± 1.8	18.1 ± 1.5
4, h		
HR, beats/min, Mean ± SD	95.1 ± 4.2	93.3 ± 12.3
MAP, mm Hg, Mean ± SD	102.1 ± 11	101.2 ± 10.3
RR, breaths/min, Mean ± SD	19.2 ± 1.5	18.7 ± 1.3
8, h		
HR, beats/min, Mean ± SD	95.2 ± 13.4	89.2 ± 13.2
MAP, mm Hg, Mean ± SD	101 ± 15.4	100 ± 14
RR, breaths/min, Mean ± SD	17.7 ± 1.2	17.2 ± 1.2
12, h		
HR, beats/min, Mean ± SD	94 ± 12.4	89.4 ± 13.3
MAP, mm Hg, mean ± SD	98.1 ± 14.2	95.4 ± 14.6
RR, breaths/min, Mean ± SD	17.1 ± 1.2	17.8 ± 1.1
16, h		
HR, beats/min, Mean ± SD	90.1 ± 13.2	86.8 ± 12.2
MAP, mm Hg, Mean ± SD	95.0 ± 12.4	93.2 ± 13.3
RR, breaths/min, Mean ± SD	16.4 ± 1.1	16.2 ± 1.1
20, h		
HR, beats/min, Mean ± SD	90.3 ± 12.3	88.2 ± 11.1
MAP, mm Hg, Mean ± SD	90.4 ± 12.2	88.3 ± 12.1
RR, breaths/min, Mean ± SD	16.5 ± 1.5	16.6 ± 1.2
24, h		
HR, beats/min, Mean ± SD	90.5 ± 11.2	88.9 ± 10.5
MAP, mm Hg, Mean ± SD	89 ± 11.1	87.1 ± 10
RR, breaths/min, Mean ± SD	15.2 ± 1.0	15.0 ± 1.1

^a^Abbreviations: HR, Heart Rate; MAP, Mean Arterial Blood Pressure; RR, Respiratory Rate; SD, Standard Deviation

^b^No significant differences were found between the groups

**Table 3.** Postoperative Pain Scores in the Gabapentin and Placebo Groups

^Gabapentin (n=25), Mean ± SD^			^Placebo (n=25), Mean ± SD^		
Variable	Sitting	Lying	Variable	Sitting	Lying
1h	35 ± 28	29 ± 25 ^a^	1h	59 ± 8	54 ± 14
4h	41 ± 17 ^a^	28 ± 16 ^a^	4h	53 ± 12	52 ± 10
8h	33 ± 15 ^a^	19 ± 9 ^a^	8h	50 ± 12	48 ± 9
12h	30 ± 12 ^a^	18 ± 10 ^a^	12h	50 ± 12	45 ± 13
16h	23 ± 9 ^a^	15 ± 8 ^a^	16h	38 ± 7	38 ± 10
20h	27 ± 12 ^a^	16 ± 8 ^a^	20h	35 ± 10	33 ± 10
24h	25 ± 7 ^a^	15 ± 7 ^a^	24h	26 ± 7	26 ± 12

^a^P < 0.05, when compared with the placebo group

**Table 4.** Morphine Consumption in the Gabapentin and Placebo Groups

	Gabapentin (n=25), Mean ± SD	Placebo (n=25), Mean ± SD
1h	5.5 ± 5.4	7.4 ± 2.7
4h	3.9 ± 3.2	8.2 ± 2.9
8h	2.8 ± 3.1 ^a^	8.1 ± 2.4
12h	2.6 ± 2.2 ^a^	9.2 ± 2.1
16h	3.43 ± 3.8 ^a^	7.15 ± 1.7
20h	2.9 ± 3.3 ^a^	6.2 ± 2.4
24h	1.2 ± 0.6 ^a^	3.5 ± 0.5
Total	33.8 ± 18 ^a^	52.45 ± 10.4

^a^P < 0.05, when compared with the placebo group

**Table 5.** Incidence of Side Effects in the Gabapentin and Placebo Groups

	Placebo (n=25)	Gabapentin (n=25)
Dizziness	1	2
Nausea	7	6
Vomiting	2	2
Somnolence	0	1
Diarrhea	2	0
Pruritus	2	0
Urinary retention	2	1
Constipation	2	2

## 5. Discussion

The main finding of this study was that a single, preoperative dose of 1200 mg gabapentin decreased total postoperative morphine consumption and postoperative pain scores at rest and upon movement after debridement of burn wounds. Moreover, gabapentin was not associated with more side effects than was the placebo. Coderre and Melzack confirmed that burn injuries not only make injured areas and surrounding tissues more painful but also cause hyperalgesia, which is a significant problem for many patients (24). In burn patients, hyperalgesia is further enhanced because the burn wounds heal slowly over days or weeks (11). Among pharmacological agents, morphine has continued to be the mainstay for both background and procedural pain management in burn patients. The long-term use of morphine has several inherent problems. Morphine binds to more than 1 receptor class, and morphine use is associated with side effects (respiratory depression, constipation, pruritus, and nausea) that limit tissue healing . An additional problem is the rapid development of tolerance to morphine and poor pain control in a small group of burn patients.Tolerance to morphine is associated with the activation of central glutamate pathways (NMDA receptors) and hyperalgesia. The main aim in combining different analgesic drugs is to obtain synergistic or additive analgesics that will allowthe administration of a smaller dose of each drug with an improved safety profile.This can be achieved by combining analgesics acting at different locations. Antihyperalgesic drugs like gabapentin may have a role in postoperative pain reduction, and their combination with other antinociceptive drugs may produce synergistic analgesic effects (25). Gabapentin is frequently used as a single preoperative oral dose for postoperative pain control in clinical studies. Single oral doses of gabapentin,as low as 600mg or 5mg/kg, have been shown to be effective in reducing postoperative pain (25). Gabapentin enhanced the analgesic effect of morphine in healthy volunteers (20), and the combination produced a better analgesic effect than morphine alone in patientswith neuropathic cancer pain (19). Gabapentin significantly decreased morphine consumption and pain in patients who underwent mastectomy (22).However, these patients were evaluated for only 4h after surgery. In our study, we found a reduction in pain scores at rest and after movement throughout the 24-h period, even though morphine consumption reduced only after the first 8h. In contrast, Fassoulaki *et al.* (26) were unable to demonstrate a decrease in analgesic consumption and VAS scores at rest or after movement during the first 24h after the operation.Differences between the results of the 2 studies can be attributed to differences in the types of surgeries performed and the types of analgesics used. Nonsteroidalanti-inflammatory drugs (NSAIDs) are useful adjunctive analgesics for decreasing pain and opioid requirements in patients undergoing major surgery. However, their use in some groups of patients may be limited by adverse renal, gastrointestinal, and hemostatic effects. In contrast, gabapentin is well tolerated and has few side effects and only minor interactions with other drugs when used for the treatment of chronic pain (26-28). We did not observe any significant side effects associated with a single oral dose of gabapentin. In conclusion, by using gabapentin preemptively as an adjuvant to morphine-based postoperative analgesia, we aimed to obtain better postoperative pain profiles with fewer narcotic-related side effects. The present study shows that a single preoperativeoral dose of 1200 mg gabapentin enhances the analgesic effect of morphine and decreases morphine consumption. However, further studies are necessary to determine the analgesic-sparing effect of different preoperative and postoperative doses of gabapentin for the management of burn pain.
